# Racial disparities in depression and mental health service use among black and white autistic adults

**DOI:** 10.1038/s41598-025-34696-8

**Published:** 2026-01-08

**Authors:** Ed-Dee G. Williams, Shalini Sivathasan, Nicole Anthony, Shaun M. Eack, Carla A. Mazefsky

**Affiliations:** 1 Boston College School of Social Work, 140 Commonwealth Ave, McGuinn Hall, Chestnut Hill, MA 02467 USA; 2 Department of Counseling, Developmental, and Educational Psychology, Boston College, Chestnut Hill, MA USA; 3 Department of Teaching and Learning, Coppin State University, Baltimore, MD USA; 4 School of Social Work and Department of Psychiatry, University of Pittsburgh, Pittsburgh, PA USA; 5 University of Pittsburgh School of Medicine, Pittsburgh, PA USA

**Keywords:** Autism, Mental health, Black/African american, Service use, Signs and symptoms, Human behaviour

## Abstract

Studies have reported that autistic individuals are diagnosed with major depressive disorder (depression) at rates significantly higher than their non-autistic peers. While studies have shown that Black autistic individuals may be particularly vulnerable to experiencing depression, few studies have examined rates of lifetime depression diagnosis and symptom burden within this population in comparison to other racial groups, in particular White autistic individuals. This study addresses this gap by comparing demographic differences and mental health diagnosis, symptoms, and service use for Black and White autistic adults with and without a lifetime depression diagnosis, offering insights to guide future research and clinical practice to address the mental health needs of autistic individuals. Data were drawn from the Relationships, Employment, Autonomy, and Life Satisfaction (REALS) study, which includes self-reported history of mental health diagnoses, as well as measures of current anxiety and depression symptoms. Bivariate analyses were conducted to examine demographic, mental health service use, and clinical differences among an age- and income-matched sample of Black and White autistic participants, stratified by whether they had received a depression diagnosis in their lifetime (past and/or current). The study included 179 autistic adults (93 Black, 86 White). Black autistic adults with a lifetime depression diagnosis had higher income, education, and employment rates than those without a lifetime depression diagnosis. White participants showed no such differences. Further, Black participants reported similarly high current depression symptoms and anxiety, regardless of whether they had a depression diagnosis or not. That is, Black autistic adults without a lifetime depression diagnosis report experiencing comparable levels of current depressive symptoms as those with a lifetime depression diagnosis, which for both groups fall near clinical cutoffs. Findings underscore the need for more nuanced mental health services that address the complex needs of autistic adults, particularly Black individuals who remain underrepresented in autism research. The similarly high anxiety and depression symptom levels across Black autistic adults with and without a lifetime depression diagnosis suggest that those with depression and who have access to mental health services may not find that such services fully address ongoing distress. The elevated rates of co-occurring mental health conditions among those with a history of depression point to the importance of integrated, intersectional approaches to care that consider both racial identity and neurodivergence.

## Introduction

Although many autistic individuals are under-assessed and underdiagnosed for major depressive disorder (depression), research shows they are significantly more likely than non-autistic peers to experience depressive symptoms, self-harm, and attempt suicide^1–3^. This risk may be even greater for Black autistic individuals, who face compounded vulnerabilities associated with the elevated rates of depression observed in the broader Black American population. However, there continues to be a lack of research focusing on depression among Black autistic individuals, whose intersecting identities of race and disability likely exacerbate their vulnerability to depression. The current study looks to expand this area of research by examining potential differences between Black and White autistic adults in the United States with and without a history of depression.

Autism is a neurodevelopmental disability characterized by differences in social communication, interaction, and comprehension. These differences can affect how individuals learn, behave, and communicate, often leading to challenges in navigating social environments, forming relationships, and adapting to changes in routine or expectations^4,5^. The presentation of these traits varies widely, making autism a highly individualized condition that requires personalized support and understanding. Beyond its clinical definition, autism is also a complex social identity. The label and traits associated with autism carry social consequences, influencing how individuals are perceived, treated, and included in society, often experiencing bullying, social exclusion, and isolation^6,7^, which is associated with increased risk of depression and anxiety. For example, Wang & Susumu (2024) conducted a systematic review of primary school-aged autistic children and found that studies have reported a significantly higher rate of bullying for autistic children compared to non-autistic children, and in some cases, even higher than children with other disabilities^8^. Similarly, Bardou et al. (2025) in a sample of 12–15-year-old autistic youth attending a variety of school types found that autistic youth attending mainstream schools reported high levels of bullying and isolation, compared to those in more autism inclusive school settings^9^.

Many of these experiences impact autistic individuals as they age and develop. Autistic adults, in general, are at a greater risk of experiencing mental health challenges than their non-autistic counterparts^2,10–13^. This elevated vulnerability is reflected in the high prevalence of co-occurring mental health conditions reported across multiple studies. Studies such as those by Lai et al. (2019) and Lugo-Marin et al. (2019) have reported the rate of co-occurring mental health disorders for autistic adults to be nearly 55%, with particularly high rates of personality and mood disorders^14,15^. Researchers have sought to better understand the specific barriers autistic adults face when attempting to access appropriate mental healthcare, given these concerning rates. Studies have found that medical providers’ misconceptions about masking (strategies used consciously or unconsciously by autistic people to hide their traits to be accepted by neurotypical people), extended wait times for mental health service use, and limited funding for treatment often hinder autistic individuals from obtaining needed support to address depressive symptoms, suicidality, and self-injurious behaviors^11^. For example, a recent study by Shaw and colleagues (2024) found that masking was associated with healthcare avoidance for autistic adults^16^. While masking may help autistic individuals navigate social situations, in healthcare settings, it can obscure symptoms, prevent accurate diagnosis, and discourage open communication. However, it is critical to acknowledge that none of these studies examined race or ethnicity, leaving unaddressed the ways systemic racism and racialized barriers may compound these challenges for autistic people of color.

Studies have found that up to 48% of autistic adults report experiencing significant depressive symptoms, with a lifetime prevalence of clinical depression being between 14 and 18%^13,17^. In addition to depression, studies have reported that autistic adults experience elevated rates of anxiety disorders compared to non-autistic peers, with estimates ranging from approximately 20% to over 40% depending on the population and methodology^12,14^. Such high prevalence contributes to substantial psychological distress, negatively impacting the quality of life for autistic adults, particularly when anxiety co-occurs with depression or other psychiatric conditions^13,15^. The presence of co-occurring anxiety and depression in autistic adults is often tied to lower life satisfaction and compromised mental health^18^. Given the close link between anxiety and depression, examining how these conditions manifest across racial groups of autistic adults is critical for understanding disparities in mental health outcomes.

For Black autistic individuals, there exist additional unique challenges to experiencing depression as well as accessing mental health support. The combination of being Black in the United States, which includes the impact of systemic racism and discrimination on health and access to care^19^, with the challenges faced by autistic individuals in an ableist society that often prevents them from living healthy and safe lives, creates an intersection of marginalized identities that uniquely impacts the lived experiences of these individuals^20,21^. This intersection significantly increases the risk of depression among Black autistic individuals. For example, studies have found that Black autistic individuals often engage in both code-switching, the act of adjusting one’s behaviors and self-presentation to mirror the norms of a dominant racial group^22^, and masking of autistic characteristics to fit into a neurotypical society, both of which are associated with increased mental distress and risk for experiencing depressive symptoms^3,23–26^. Benedetto (2024), focusing specifically on Latina autistic individuals, introduced the concept of the “dual masking phenomenon,” in which autistic individuals mask not only their autistic traits but also aspects of other marginalized identities they hold^27^. Williams et al. (2023), a year prior, described similarly how Black autistic individuals may engage in both masking their autistic traits and code-switching to navigate racialized environments^25^. This overlapping process can intensify psychological distress, increasing the risk of experiencing depression. Further compounding these challenges, recent research on community mental health service use shows that Black autistic adolescents and young adults are significantly less likely than their White peers to receive diagnoses for common mental health conditions or access community-based mental health care. These gaps persist even after accounting for socioeconomic and clinical factors. Such disparities point to systemic barriers and underscore the urgent need for greater equity-focused research and interventions^28^.

Furthermore, studies have found that Black autistic individuals often report limited access to adequate physical and mental health support^23,29^, thus preventing them from accessing critical services to address symptoms of depression. For example, one study examined the well-being of a small sample (*N* = 39) of Black and White autistic youth transitioning to adulthood. Despite both groups receiving depression diagnoses at equal rates, researchers found that Black autistic youth reported significantly higher levels of depressive symptoms compared to White autistic youth^25^.

Lack of access to treatment is troublesome, given the heightened risk of Black autistic individuals experiencing depression or depressive symptoms. Not only is it essential to understand how depression is experienced by Black autistic individuals and barriers to mental health services, but it is also key to begin identifying the particular demographic factors that may make Black autistic individuals more vulnerable to depression than their White counterparts. Therefore, the current study sought to examine potential demographic differences among Black and White autistic adults who report a history of depression diagnosis, with those without a lifetime history of depression diagnoses.

### Research question and approach

This study was guided by the following research question: How do Black autistic adults with and without a lifetime history of depression diagnosis differ in demographics, mental health symptoms, and service utilization, compared to White autistic adults with and without a history of depression? We hypothesized that overall, White autistic adults would be more likely to have access to mental health diagnoses and subsequent interventions compared with Black autistic adults. As such, we specifically predicted that Black autistic adults with a lifetime history of depression diagnosis would report lower levels of mental health service utilization and exhibit greater current mental health symptom burden, particularly elevated depressive and anxiety symptoms, compared to White autistic adults with a similar diagnostic history. Additionally, we expected that Black autistic adults, regardless of depression diagnosis status, would report significant levels of depression symptom burden.

### Methods

The University of Pittsburgh Institutional Review Board approved all study procedures, and all methods were conducted in accordance with relevant guidelines and regulations. Informed consent was obtained from all subjects and/or their legal guardian(s) before participating in the study. Data for this study come from the Relationships, Employment, Autonomy, and Life Satisfaction (REALS) study^30,31^, which aimed to develop a new self-report questionnaire to measure various aspects of adult life in autistic adults and adults with intellectual and developmental disabilities (IDD). Adults ≥ 18 years were invited to complete a battery of measures online through the SPARK Research Match registry (Feliciano et al., 2018) and via ads to local, state, and national autism and IDD groups and registries. SPARK represents the largest online research registry of individuals (and their families) living in the United States who report having a formal autism diagnosis made by a healthcare professional, matching these individuals to various research studies in which they can participate.

### Measures used in the current study

#### Sociodemographic survey

Autistic participants completed a sociodemographic survey, which included providing their age, racial and ethnic identity, gender, personal income, and educational attainment, as well as their lifetime history of mental health diagnoses and current and lifetime service utilization.

#### PROMIS emotional distress measures for depression and anxiety

The Patient-Reported Outcomes Measurement Information System-Emotional Distress, Short Form (PROMIS-SF) Depression subscale for adult self-report was used in the current study to capture current depression symptoms over the past 7 days^32^. Given the close association between depression, anxiety, and mental distress (Uljarevic et al., 2021; Murray et al., 2019)^33,34^, the PROMIS-SF Anxiety subscale was also included in the analysis as an additional measure of emotional distress. T-scores > 55 are suggestive of clinically significant symptom impairment. Both scales have demonstrated strong internal consistency in use with autistic adults^35^.

### Participant sample and data analysis plan

Participants from the larger REALS study who identified “Black/African American” as at least one of their racial identities, and an age- and income-matched sample of those who identified only “White” as their racial identity, were included in the analytic sample for the current study. These participants were further divided into two groups - those who endorsed having a lifetime history of a depression diagnosis (i.e., current or previous depression diagnosis, combined here as “Hx Depression”) and those who endorsed no history of depression diagnosis (“No Hx Depression”).

We employed bivariate analysis, including t-tests and chi-square analyses, to compare Hx Depression and No Hx Depression participants within racial groups across the described demographic variables (age, racial and ethnic identity, gender, personal income, and educational attainment, employment status), mental health diagnoses, current symptoms, and service use.

## Results

### Sociodemographics

A total of 179 autistic adults had complete data for the lifetime depression diagnosis grouping variable and were thus included in the current study (*n* = 93 Black/African American, *n* = 86 White). The average age within this matched sample was *M* = 31 years (*SD* = 9 years), t(177) = 0.17, *p* =.75, and there were no significant differences in income distribution, *X*^2^(7) = 5.67, *p* =.58, with the majority (85%) of the total sample reporting personal incomes below $66,000.

#### Within Racial identity group comparisons

See Table 1 for within-group sociodemographic comparisons. Among White autistic adults, there were no significant differences between the Hx Depression and No Hx Depression groups in terms of age, income, gender, educational attainment, or proportion currently employed (all *ps* > = 0.05). In contrast, there were several significant sociodemographic differences between Black autistic adults in the Hx Depression and No Hx Depression groups. Black autistic adults in the Hx Depression group had significantly higher income and educational attainment, and were more likely to be working full time, than those in the No Hx Depression group (*ps* < 0.01). Additionally, Black autistic adults in the Hx Depression group were slightly older (*M*_age_=34 years) than those in the No Hx Depression group (*M*_age_=27 years) (*p* =.048). Finally, there was a difference in gender distribution among Black autistic adults with and without depression (*p* =.02), such that 60% of participants in the No Hx Depression group selected their gender as ‘male’ (33% as ‘female’, 7% as another gender), whereas 51% the Hx Depression group identified as ‘female’ (24% as ‘male’, 16% as another gender).

**Table 1:** Total participant sociodemographics and group by history of depression status.

	Black Autistic Adults (*n* = 93)			White Autistic Adults (*n* = 86)		
	No Hx Depression *n* = 30	Hx Depression *n* = 63	*p*	No Hx Depression *n* = 36	Hx Depression *n* = 50	*p*
Age in years, mean (SD)	26.53 (6.82)	33.77 (8.87)	0.048	30.04 (8.65)	32.05 (8.94)	0.67
Gender, n (%)						
Male/Man	18 (60%)	15 (23.8%)		21 (58.3%)	17 (34%)	
Female/Woman	10 (33.3%)	32 (50.8%)		14 (38.9%)	25 (50%)	
Nonbinary/Genderfluid	2 (6.7%)	10 (15.9%)	0.02	1 (2.8%)	6 (12%)	0.14
Unsure/Questioning	0 (0%)	2 (3.2%)		0 (0%)	1 (2%)	
Other	0 (0%)	2 (3.2%)		0 (0%)	1 (2%)	
Prefer not to say	0 (0%)	2 (3.2%)		0 (0%)	0 (0%)	
Multi-racial identification, n (%)						
Asian American	4 (13.3%)	0 (0.0%)	0.009			
American Indian/Alaska Native	6 (20%)	5 (7.9%)	0.17			
Native Hawaiian/Pacific Islander	0 (0%)	0 (0%)	-	-	-	-
White	10 (33.3%)	19 (30.2%)	0.76			
Another race	2 (6.7%)	0 (0.0%)	0.1			
Ethnicity, n (%), *n* = 92						
Hispanic/Latino	2 (7.7%)	12 (20.3%)	0.15	3 (8.3%)	8 (16%)	0.29
Non-Hispanic/Non-Latino	24 (92.3%)	47 (79.7%)		33 (91.7%)	42 (84%)	
Personal income, n (%)						
Less than $20,999	14 (46.7%)	8 (12.7%)		12 (33.3%)	12 (24%)	
$21,000 to $35,999	12 (40%)	16 (25.4%)		11 (30.6%)	16 (32%)	
$36,000 to $50,999	0 (0%)	14 (22.2%)	0.004	5 (13.9%)	6 (12%)	0.77
$51,000 to $65,999	2 (6.7%)	5 (7.9%)		5 (13.9%)	9 (18%)	
$66,000 to $80,999	2 (6.7%)	4 (6.3%)		0 (0%)	4 (8%)	
$81,000+	0 (0%)	12 (19%)		2 (5.6%)	3 (6%)	
Don’t know/Prefer not to say	0 (0%)	4 (6.4%)		0 (0%)	0 (0%)	
Educational Attainment, n (%)						
Up to high school diploma	12 (40%)	6 (9.5%)		8 (22.2%)	3 (6%)	
Some college/technical school	10 (33.3%)	24 (38.1%)	0.0008	10 (27.8%)	20 (40%)	0.15
Bachelor’s degree	6 (20%)	10 (15.9%)		12 (33.3%)	18 (36%)	
Some post-graduate/post-grad degree	2 (6.7%)	23 (36.5%)		6 (16.7%)	9 (18%)	
Employment Status, n (%)						
Working full time	2 (6.7%)	24 (38.1%)		18 (50%)	21 (42%)	
Working part-time	8 (26.7%)	22 (34.9%)	0.0002	7 (19.4%)	15 (30%)	
Unpaid student intern	0 (0%)	0 (0%)		1 (2.8%)	1 (2%)	0.72
Unemployed/looking for work	12 (40%)	4 (6.3%)		6 (16.7%)	5 (10%)	
Unable to work	8 (26.7%)	12 (19%)		4 (11.1%)	7 (14%)	
Retired	0 (0%)	1 (1.6%)		0 (0%)	1 (2%)	

*Note. All participants in the current study identified as Black/African American, some of whom also endorsed additional multiracial/ethnic identities shown here.

### Mental health diagnoses and current symptoms

Overall, there were no statistically significant differences between what the Black autistic group and their age- and income-matched White autistic group reported regarding lifetime mental health diagnoses. Both groups reported comparably high rates of depression (i.e., Hx Depression groups; 68% and 58%, respectively, *X*^2^(1) = 1.77, *p* =.18) and anxiety diagnoses (75% vs. 71%, *X*^2^(1) = 2.95, *p* =.09), followed by attention deficit hyperactivity disorder (ADHD) (54% vs. 48%, *X*^2^(1) = 0.51, *p* =.47), post-traumatic stress disorder (PTSD) (42% vs. 30%, *X*^2^(1) = 2.95, *p* =.09), obsessive-compulsive disorder (OCD) (35% vs. 37%, *X*^2^(1) = 0.06; *p* =.81), and bipolar disorder (27% vs. 21%, *X*^2^(1) = 1.03; *p* =.31. Furthermore, as a whole, Black and White autistic participants did not differ in their levels of current anxiety (*p* =.13), or depression (*p* =.45) symptoms as reported on PROMIS measures.

#### Within Racial identity group comparisons

**Fig. 1:**
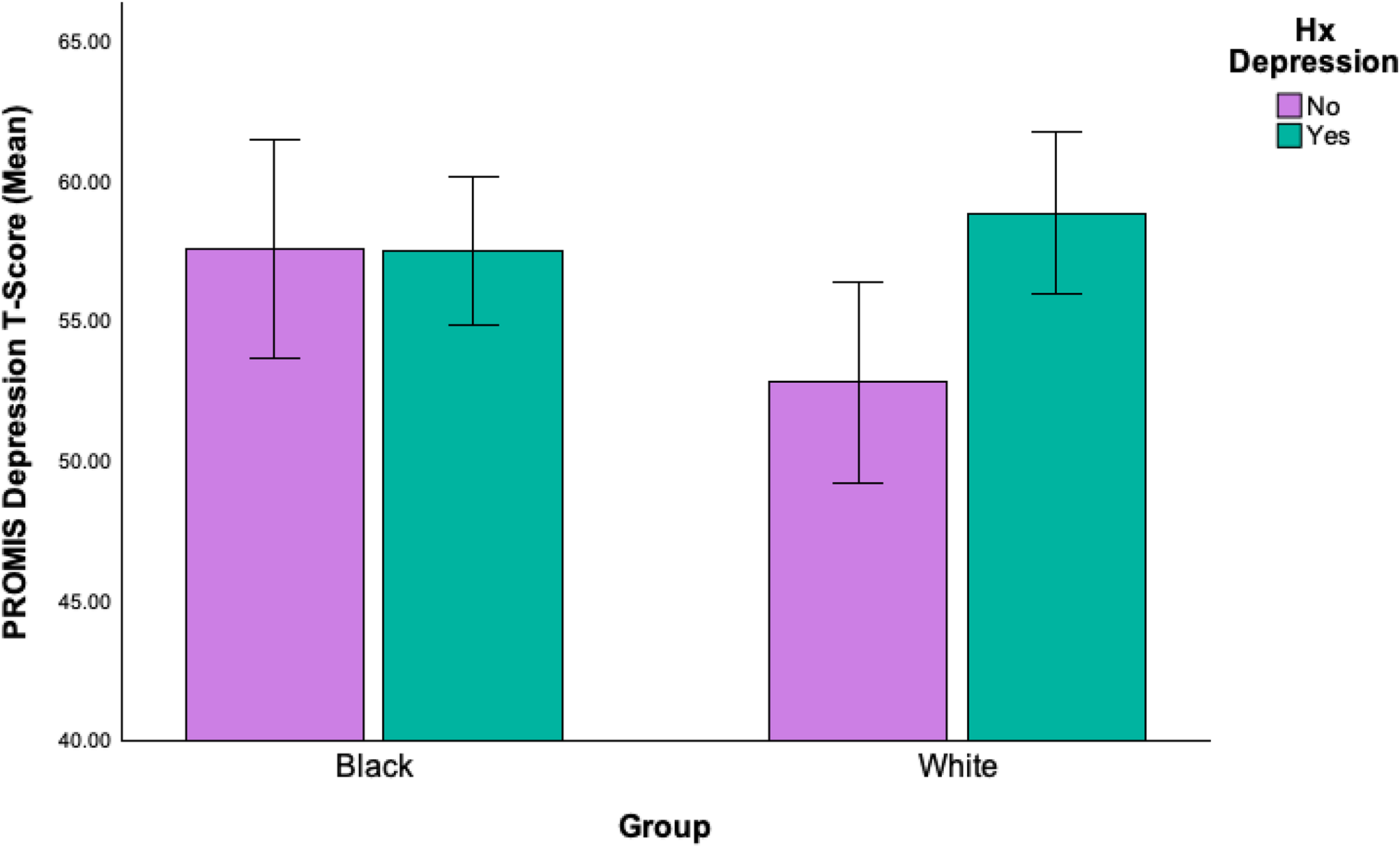
Group x lifetime depression diagnosis on current depression symptoms.

See Table 2 for within-group comparisons of co-occurring mental health diagnoses and service use between Black and White autistic participants with and without a lifetime depression diagnosis. For both racial groups, autistic adults in the Hx Depression Groups were significantly more likely to also have reported a lifetime history of co-occurring anxiety, bipolar disorder, OCD, PTSD, and ADHD diagnoses (all *ps* < = 0.05). In terms of current anxiety and depression symptoms, however, within-group analyses revealed that while White autistic participants in the Hx Depression group had significantly higher depression and anxiety ratings than White participants in the No Hx Depression group, this pattern was not found for Black autistic participants (see Fig. 1). Specifically, Black autistic adults in the Hx Depression and No Hx Depression groups report experiencing comparable levels of current depressive symptoms, which for both groups fall near clinical cutoffs on the PROMIS measure (i.e., T-Score = 60).

**Table 2:** Mental health diagnosis, symptoms, and service use.

	Black Autistic Adults (*n* = 93)			White Autistic Adults (*n* = 86)		
	No Hx Depression	Hx of Depression	*p*	No Hx Depression	Hx of Depression	*p*
Co-occurring diagnoses (lifetime), n (%)						
Anxiety Disorder, *n* = 174	10 (35.7%)	57 (90.5%)	< 0.0001	15 (44.1%)	43 (87.8%)	< 0.0001
Bipolar Disorder = 175	4 (14.3%)	22 (34.9%)	0.05	2 (5.6%)	16 (33.3%)	0.003
OCD, *n* = 173	4 (13.3%)	30 (49.2%)	0.001	7 (19.4%)	23 (50%)	0.006
PTSD, *n* = 178	6 (20%)	34 (54.0%)	0.003	5 (13.9%)	21 (42.9%)	0.005
ADHD, *n* = 176	10 (33.3%)	39 (61.9%)	0.014	11 (31.4%)	28 (58.3%)	0.025
PROMIS-SF (current symptoms), mean (SD)						
Anxiety T-Score, *n* = 164	58.52 (10.6)	61.89 (11.3)	0.2	55.25 (10.8)	60.56 (9.7)	0.026
Depression T-Score, *n* = 162	57.58 (9.7)	57.49 (11.1)	0.97	52.78 (8.4)	58.84 (10.0)	0.006
**Current Medication Use**,** n (%)**						
Antipsychotics, *n* = 110	0 (0%)	6 (14.6%)	0.17	1 (6.3%)	4 (10.8%)	1
Antidepressants, *n* = 115	4 (25%)	32 (71.1%)	0.002	8 (50%)	22 (57.9%)	0.77
Mood Stabilizer, *n* = 114	4 (25%)	13 (28.9%)	1	4 (25%)	14 (37.8%)	0.53
Psychostimulant, *n* = 113	6 (37.5%)	12 (26.7%)	0.53	3 (18.8%)	10 (27.8%)	0.73
Current Psychiatric Service Use, n (%)						
Individual, *n* = 179	12 (40%)	32 (50.8%)	0.38	10 (27.8%)	27 (54%)	0.03
Group, *n* = 179	2 (6.7%)	8 (12.7%)	0.49	0 (0%)	1 (2%)	1
Behavioral, *n* = 179	6 (20%)	8 (12.7%)	0.37	3 (8.3%)	6 (12.0%)	0.73
Lifetime psychiatric service use, n (%)						
Individual, *n* = 179	18 (60.0%)	57 (90.5%)	0.001	14 (38.9%)	37 (74.0%)	0.002
Group, *n* = 179	4 (13.3%)	20 (31.7%)	0.077	3 (8.3%)	15 (30.0%)	0.017
Behavioral, *n* = 179	14 (46.7%)	24 (38.1%)	0.5	12 (33.3%)	22 (44.0%)	0.38
Lifetime psychiatric hospitalizations, n (%), *n* = 179						
0 hospitalizations	20 (66.7%)	31 (49.2%)		28 (77.8%)	19 (38%)	
1 hospitalization	4 (13.3%)	14 (22.2%)	0.19	4 (11.1%)	15 (30%)	0.003
2–4 hospitalizations	6 (20%)	12 (19%)		2 (5.6%)	11 (22%)	
5 + hospitalizations	0 (0%)	6 (9.5%)		2 (5.6%)	5 (10%)	

Note. Independent t-tests, Pearson chi-square (with fisher exact test used for cells with values < 5).

### Mental health service use

Similar to overall mental health diagnosis rates, there were no significant differences between Black and White autistic participants in terms of overall psychiatric medication use, lifetime hospitalizations, and current or lifetime behavioral service use (all *ps* > 0.05), with a few exceptions (see Table 2). Black autistic participants overall reported a greater likelihood of accessing individual therapy (lifetime), *X*^2^(1) = 12.38, *p* <.0001, and group therapy (current), *X*^2^(1) = 7.05 *p* =.01, compared with White autistic participants.

#### Within Racial identity group comparisons

Within-group comparisons reveal a more complicated picture: White autistic participants in the Hx Depression group were more likely to report accessing individual therapy (current and lifetime), group therapy (lifetime), and psychiatric hospitalizations than those in the No Hx Depression group (all *ps* < 0.05). In contrast, there were no differences for Black autistic participants in the Hx and No Hx Depression groups in use of individual therapy (lifetime) group therapy (current and lifetime), and psychiatric hospitalization (*ps* > 0.05); Black autistic participants in the Hx Depression group were more likely to report using individual therapy in their lifetimes (lifetime) compared to the No Hx Depression group (*p* =.001). When it comes to medication use, White participants were equally likely to report antidepressant use regardless of their lifetime history of depression diagnosis (*p* =.77), whereas Black autistic participants in the No Hx Depression group were significantly less likely to access antidepressants than Black autistic participants in the Hx Depression group (*p* =.002). Said another way, a greater proportion of Black autistic adults without a depression diagnosis report utilizing outpatient and inpatient services, but less antidepressant medications, than age- and income-matched White autistic adults.

## Discussion

This study aimed to explore how Black autistic adults, with and without a lifetime history of depression diagnosis, differ from an age- and income-matched sample of White autistic adults with and without a depression diagnosis in demographics, co-occurring mental health conditions, and service utilization. We hypothesized that White autistic adults would be more likely than their Black counterparts to access mental health diagnoses and subsequent interventions. Additionally, we predicted that Black autistic adults with a lifetime history of depression diagnosis would report less service utilization and exhibit greater current mental health symptoms than White autistic adults with a lifetime history of depression diagnosis.

As a whole, when matched by age and income, White and Black autistic adults reported comparable rates of lifetime depression, anxiety, ADHD, PTSD, OCD, and bipolar diagnoses, and current anxiety and depressive symptoms. However, stark differences emerged when comparing those who had received a depression diagnosis and those who did not *within* racial groups, highlighting the importance of considering intersectionality of various identities within and between racial groups. The findings revealed notable sociodemographic differences among Black autistic adults based on their lifetime depression history, but not among their White counterparts. Black autistic adults with a lifetime history of depression diagnosis (Hx Depression) tended to have higher incomes, greater educational attainment, and were more likely to be employed full-time compared to those without such a history (No Hx Depression). These patterns suggest that socioeconomic status (SES) may significantly influence access to mental health diagnosis in this group, but not among White autistic adults. Higher SES, often linked to better access to healthcare, could increase the likelihood of receiving a formal depression diagnosis and accessing treatment. At the same time, navigating predominantly White, neurotypical, or high-pressure environments may contribute to elevated psychological distress, potentially raising the risk of depression for those employed full-time in higher-paying jobs.

Additionally, Black autistic adults in the Hx Depression group were on average 7 years older and were less likely to identify as male (24%) than those in the No Hx Depression group (60% male). The finding suggests potential age- and gender-related factors in the development or reporting of depressive symptoms for Black autistic individuals. One possible explanation is that older Black participants may have had more time or opportunities to receive a formal diagnosis or to experience depressive episodes, reflecting a cumulative exposure to stressors over time. Alternatively, this age difference may indicate generational disparities in mental health awareness, access to care, or willingness to disclose psychological distress for Black autistic individuals. In regard to gender, existing research on non-autistic populations suggests that gender plays a vital role in mental health experiences and help-seeking behaviors among Black individuals. Studies have found that Black men are often underdiagnosed for depression despite reporting higher levels of depressive symptoms compared to Black women^36^. In contrast, Black women are more likely to utilize available mental health services than Black men^37^. These patterns point to gendered differences in stigma, help-seeking, and access to care. However, there is currently no research examining whether similar disparities exist among Black autistic men and women. This gap underscores the need for future studies to explore gender-specific experiences within this population to inform culturally and contextually responsive interventions.

In contrast, no significant sociodemographic differences were found between White autistic adults with and without a history of depression. This may indicate more consistent access to mental health care across SES levels and age in this group. Alternatively, it may reflect different cultural or systemic factors affecting how symptoms are both reported to and understood by clinicians, and ultimately whether a depression diagnosis is made. Overall, the results point to potential disparities in how depression is experienced and diagnosed among autistic adults across racial and socioeconomic lines. These findings align with studies of neurotypical individuals, which have found that Black people are often underdiagnosed with depression while reporting higher rates of depressive symptoms, report limited access to mental health services, and underutilize available mental health resources^38–40^.

Existing research examining depression among autistic individuals by race is limited but emerging. Most existing studies either fail to report race-specific outcomes or include samples of racial minorities that are too small to yield meaningful conclusions^41^. As a result, autistic individuals of color remain largely overlooked in mental health research. However, some studies have documented preliminary findings of racial disparities in psychiatric diagnoses among autistic adults, with Black and other minority groups less likely to receive formal diagnoses compared to White peers^42^. Initial evidence also suggests that Black autistic youth may experience depressive symptoms at rates comparable to or higher than White autistic youth^25^, despite facing greater barriers to care^28^. Other work highlights that autistic traits may be more strongly associated with depression and anxiety among Black individuals than among White individuals^43^, pointing to unique risk factors at the intersection of race and autism. Collectively, these findings underscore the importance of situating our results within a broader context of racial inequities in mental health and the urgent need for research that explicitly addresses depression among autistic adults across racial groups.

This gap makes it difficult to determine whether observed disparities reflect broader patterns or unique challenges at the intersection of autism and race. Our findings begin to address this gap by showing that Black autistic adults without a depression diagnosis report symptom severity comparable to those with a diagnosis, suggesting that underdiagnosis may be a significant concern. Situating these results within the context of racial inequities in mental health care underscores the need for research that explicitly explores depression among autistic adults across racial groups and identifies culturally responsive strategies to improve assessment and treatment.

Consistent with prior research, our findings indicate that among White autistic participants, those with a lifetime depression diagnosis reported significantly higher levels of current depressive and anxiety symptoms compared to those without such a history. Their scores on the PROMIS mental health measures align with their diagnostic history (i.e., on average below clinical cutoffs in the No Hx Depression group, and at/above clinical cutoffs in the Hx Depression group). However, for Black autistic participants in the No Hx and Hx Depression groups, their current self-reported symptoms were nearly identical. Notably, Black participants in both groups reported levels of depression symptoms that hovered around the clinical cutoff. In other words, Black autistic adults who had never received a depression diagnosis were experiencing symptoms just as significantly as those who had. This finding suggests that many Black autistic adults may be living with undiagnosed depression, as their symptom severity mirrors that of diagnosed peers. The absence of diagnostic differentiation highlights systemic gaps in recognition and assessment.

Factors such as systemic bias, cultural stigma, or limited access to culturally competent mental health care may all play a role. Meanwhile, the more consistent pattern seen among White autistic adults, where depression diagnosis aligns with symptom severity, highlights a disparity in how mental health conditions are identified and treated across racial lines. These findings underscore the urgent need for more equitable, inclusive, and culturally responsive approaches to mental health assessment and care in autistic communities.

The present findings additionally reveal important racial disparities in mental health service utilization among autistic adults, not just in relation to depression diagnosis but also in treatment. While overall use of psychiatric medications such as antipsychotics, mood stabilizers, and stimulants was comparable across racial and diagnostic groups, notable differences emerged in the use of antidepressants and non-pharmacological services. Antidepressant use among White autistic adults was consistently high, regardless of diagnosis, suggesting broader access or more proactive prescribing. In contrast, Black autistic adults without a diagnosis were significantly less likely to use antidepressants, despite reporting similar symptom severity. This disparity may reflect systemic barriers such as provider bias, cultural stigma, and limited access to culturally competent care.

Masking may be a key factor influencing both symptom recognition and access to services, as well as being associated with stigma and systemic discrimination. For Black autistic adults, masking can lead to underreporting of emotional distress and misinterpretation by providers, especially in high-pressure environments where conformity to neurotypical and racialized norms is expected. As noted by Pearson & Rose (2021), Shaw et al. (2024), and Evans et al. (2024), masking can obscure mental health struggles, resulting in a preference for pharmacological interventions over therapeutic ones^5,24,44^. The findings also reinforce perspectives from Williams et al. (2022) and Benedettos’ (2024) dual masking theory, in which autistic individuals of color take part in both masking and code-switching behaviors, potentially correlating with higher levels of mental distress as well as greater disparities in mental health diagnosis and accessing needed services^25,27^. For Black autistic individuals, it is possible that the high rate of depressive symptoms among those without a formal depression diagnosis is associated with greater masking behaviors that may also be masking depressive symptoms.

Patterns of non-medication service use further underscore these disparities. White autistic adults with a depression diagnosis were significantly more likely to report lifetime use of individual therapy, group therapy, and psychiatric hospitalization compared to those without a diagnosis. This suggests that, for White participants, a depression diagnosis is closely linked to increased engagement with mental health services. However, among Black autistic adults, service utilization was largely similar regardless of diagnostic status, with the exception of individual therapy, which was more commonly reported by those with a diagnosis. Notably, Black autistic adults without a depression diagnosis reported rates of outpatient and inpatient service use that were comparable to or higher than those of age- and income-matched White peers. This aligns with previous studies of non-autistic Black adults that have reported that Black adults often overly rely on emergency room services and primary care for mental health and psychiatric support^45,46^.

### Limitations and future directions

While the current study provides crucial initial insights into potential disparities among Black and White autistic individuals with and without a lifetime history of depression diagnosis, it is essential to acknowledge several limitations. First, the study relies on a relatively small and non-representative sample of research-engaged participants; therefore, the findings may not be generalizable to the larger autistic population. Furthermore, participants recruited from the SPARK match registry tend to report higher levels of educational attainment and higher average incomes than the larger autism population, potentially skewing results. Future research should explore similar demographic differences using a nationally representative sample to validate the reported findings.

Additionally, the secondary data used in this study is largely based on a sociodemographic self-report survey, lacking clinical verification of depression history or current diagnoses, and does not provide a comprehensive list of alternate diagnoses (e.g., schizophrenia) or symptom profiles (e.g., suicidality or self-harm behaviors). Furthermore, incorporating additional variables related to interpersonal experiences, such as encounters with racism, discrimination, minority stress, and navigating mental health services, could enhance our understanding of depression outcomes and service utilization. Furthermore, future studies should examine the role of age and gender more closely, as the current study identified both as significant demographic factors.

Finally, due to its cross-sectional design, the study cannot establish causal relationships between demographic factors and depression outcomes. Longitudinal data would be valuable for identifying both causality and the direction of associations between different demographics and depression in Black and White autistic individuals.

## Conclusion

Findings from this study reveal a critical disconnect between symptom severity, mental health diagnosis, and service use among Black autistic adults with and without a lifetime history of depression diagnosis, compared to their White counterparts with and without such a history. Despite reporting depressive symptoms near clinical thresholds, many Black autistic adults remain undiagnosed and access fewer psychiatric medications, even while engaging with mental health services. This suggests that diagnosis may not be functioning as a reliable gateway to care in this population, raising concerns about the equity and effectiveness of current diagnostic and treatment practices.

The study also underscores the importance of examining within-group differences among Black autistic individuals. While cross-group comparisons are valuable for identifying disparities, they risk reinforcing deficit-based narratives if not complemented by analyses that center the diverse and intersectional experiences within marginalized populations. Future research should prioritize the unique needs of Black autistic individuals, moving beyond homogeneity assumptions to develop culturally responsive and tailored supports that address systemic barriers in mental health care.

Together, these findings highlight the urgent need for more equitable, inclusive, and culturally competent approaches to mental health assessment and intervention in autistic communities, particularly for those at the intersection of racial and neurodivergent identities.

## Data Availability

The datasets analyzed during the current study are not publicly available, as this ongoing study is being used to develop a new measure of aspects of real adult life in autistic adults and adults with intellectual and developmental disabilities (IDD). However, requests for the data can be made to the project coordinator at AFSstudy@upmc.edu.
